# Animal Welfare in Colombian Poultry Industry: Exploring Veterinarians’ Perceptions, Attitudes, Knowledge, Barriers, and Professional Profiles

**DOI:** 10.1155/vmi/3976972

**Published:** 2026-10-06

**Authors:** Laura García-Naranjo, Marlyn H. Romero

**Affiliations:** ^1^ Facultad de Ciencias Agropecuarias Universidad de Caldas Manizales 170004 Colombia ucaldas.edu.co

**Keywords:** animal welfare, poultry industry, professional profiles, veterinarians

## Abstract

Animal welfare (AW) in Colombian commercial poultry farming represents a growing challenge in which veterinarians and animal scientists play a strategic role as agents of technical transfer between the regulatory framework and production practices. This study aimed to characterize the knowledge, attitudes, and perceptions regarding AW; identify the main barriers, limitations, and opportunities for improvement perceived by professionals involved in Colombian commercial poultry farming; and construct professional profiles that synthesize the heterogeneity of these dimensions. A quantitative, descriptive, and cross‐sectional study was conducted using a structured online survey administered to 106 professionals. Quantitative data were analyzed through descriptive statistics, principal component analysis (PCA), multiple correspondence analysis (MCA), and hierarchical cluster analysis using Ward’s method. The results revealed predominantly favorable attitudes toward AW and a strong recognition of its productive implications, although gaps were found in the conceptual understanding of the construct and a lack of awareness of specific regulatory aspects. The main perceived barriers were producers’ lack of economic motivation, resistance to change, and beliefs about decreased productivity. Four statistically distinct professional profiles were identified: technical welfare leaders (25.5%), convinced by structural barriers (34.9%), professionals with relational barriers (15.1%), and results‐oriented pragmatists (24.5%). The similarity in knowledge, attitude, and perception scores among profiles indicates that professional heterogeneity is primarily determined by the perception of barriers and opportunities, rather than by the level of knowledge. These findings suggest relevant areas for further investigation and may help inform the development of future intervention strategies for the implementation of AW standards in the Colombian poultry sector.

## 1. Introduction

The intensification of animal production has heightened public awareness regarding environmental conservation, food safety, and animal welfare (AW), with the aim of promoting food safety, food security, and sustainable food production [1, 2]. Additionally, shifts in consumer preferences and advances in scientific knowledge regarding the direct relationship between AW and production performance have led numerous countries to update their legislation and establish sanitary standards [3]. Colombian legislation recognizes animals as sentient beings [4] and has regulated the welfare conditions for commercial poultry, including broilers and laying hens [5]. This regulation establishes evaluation methodologies focused on key aspects such as environmental control and animal‐based health measures [6]. Furthermore, the Ministry of Agriculture and Desarrollo Rural created and regulated the National Council and the National Technical Committee on AW for production animals [7]. These entities were established with the mandate of safeguarding AW standards. Simultaneously, they ensure alignment with sanitary and food safety requirements across all stages of the productive chain [7].

The relationship between poultry welfare and veterinary practice is particularly close [8]. Veterinary professionals are fundamental agents in the transmission of scientific and regulatory standards to producers [9]. Their role as agents of change is central to fostering attitudes of continuous improvement, regulatory updating, and the application of technical knowledge in support of poultry health and welfare [10]. Likewise, their capacity to influence the perceptions and practices of producers makes their ethical and professional formation a strategic factor for the sustainable development of the sector [11, 12]. Accordingly, the perceptions, knowledge, and attitudes of veterinary professionals constitute a critical interface between scientific evidence and practical application, directly influencing decision‐making regarding AW [13]. Nevertheless, although professionals are expected to act as proactive advisors, they frequently encounter difficulties in translating this awareness into daily practice; several studies have highlighted the need to strengthen their advisory approach in order to inspire behavioral change among producers [14, 15]. Prior research has also shown that producers’ trust in professionals’ knowledge and experience, as well as professionals’ communication skills, are important determinants of successful implementation [16, 17]. However, some studies have found no consensus between poultry farmers and professionals regarding the definition of AW, the interpretation of welfare indicators, and their practical utility [18]. These discrepancies may affect the timely detection of AW problems, the formulation of control strategies, and overall decision‐making [19].

A lack of understanding of the factors that influence professionals’ adoption behavior regarding welfare standards may compromise the level of legislative implementation, particularly in countries where AW education is still emerging, as is the case in Colombia [20]. Poultry welfare is a multidimensional concept that spans all stages of the production chain, including on‐farm rearing, handling, live transport, and slaughter [10, 21]. Each phase presents specific challenges such as thermal stress, stocking density, physical trauma, and preslaughter management that can directly compromise bird health, product quality, and overall production efficiency [10, 22]. Understanding professional perceptions across these diverse operational stages is therefore essential for implementing comprehensive welfare strategies. An additional relevant factor concerns differences in perception and attitude according to: (a) professional experience and gender, (b) employment status, (c) sociodemographic characteristics, and (d) educational background, among other aspects [8, 23]. Moreover, AW is not a uniform variable; its interpretation depends on both normative and institutional contexts, as well as on the internal cultural frameworks of the veterinary community [10]. Although technical knowledge is essential, evidence indicates that it does not automatically translate into the effective adoption of AW practices [24]. Beyond objective information, professionals’ personal ethical attitudes and risk perceptions play a decisive role in driving action; they influence how technical guidelines are prioritized, interpreted, and implemented during daily decision‐making on farms [14, 24]. Consequently, positive attitudes and a strong ethical commitment are often as influential as technical expertise in ensuring that welfare standards are meaningfully applied in practice [14, 24].

Currently, there is growing research interest in understanding the motivations and barriers that contribute to, or hinder, the implementation of AW practices by both professionals and producers [25, 26]. Recent studies have employed knowledge, attitude, and perception surveys; personal interviews; and focus group discussions [11, 13] to explore the perspectives of poultry farmers and veterinary professionals in relation to AW. These studies highlight key strategies to promote the implementation of AW standards, such as integrating continuous sensor‐based monitoring technologies, adopting communication techniques like motivational interviewing, and utilizing interactive digital tools for welfare assessment [11, 15, 27]. These approaches help clarify the motivations behind welfare improvements and bridge the gap between AW research and its practical application in the field [11]. Given that Colombia lacks studies in this area specifically directed at veterinary professionals and/or animal scientists working in the poultry sector, the objectives of this study were as follows: (a) to assess professionals’ perceptions of AW, including perceived barriers, limitations, and opportunities for improvement; (b) to evaluate their knowledge and attitudes regarding AW; and (c) to identify professional profiles based on these characteristics. Understanding these dimensions may provide valuable information for identifying areas of improvement in professional practice and for designing effective strategies for the implementation and enforcement of current welfare regulations.

## 2. Materials and Methods

### 2.1. Ethical Considerations

This research was reviewed and approved by the Ethics and Animal Experimentation Committee of the Faculty of Agricultural Sciences of the University of Caldas under the minimal risk research category (Approval No. FCA‐2024/Act‐03; date: December 5, 2024). All procedures involving human participants were conducted in accordance with the ethical principles established in the Declaration of Helsinki and relevant institutional guidelines. Written informed consent was obtained from all participants prior to their inclusion in the study, including consent for the use of their anonymized responses for research and publication purposes. The anonymity and confidentiality of all participant information were guaranteed throughout the study. Prior to completing the survey, participants were fully informed of the study objectives, methods, and potential implications for both themselves and the field of study and were explicitly notified of their right to decline to answer any question or to withdraw from the study at any time without consequence.

### 2.2. Description of the Study

A descriptive, cross‐sectional quantitative study was conducted as part of a broader research initiative that incorporated a sequential design including semistructured interviews (qualitative component). This manuscript exclusively reports the results of the quantitative phase, consisting of an online survey administered via Google Forms to veterinarians and/or animal scientists working in the poultry sector. This phase was conducted in Colombia from August 10 to December 27, 2025.

A structured survey instrument was developed incorporating both original items and items adapted from previously validated instruments in the field [21], designed to assess the knowledge, attitudes, and perceptions of professionals working in commercial poultry production including broiler and laying hen systems regarding AW, as well as the barriers, opportunities for improvement, and limitations perceived for the implementation of AW standards. A pilot test was conducted by administering the survey to 10 professionals to evaluate the clarity and relevance of the items; the participants confirmed that the questions were clear and comprehensible, so no modifications were required to the survey instrument. These responses were excluded from the final analysis. A nonprobabilistic sampling approach combining convenience and chain‐referral (snowball) sampling was employed. Initial contact was made with professionals affiliated with the Colombian Association of Poultry Veterinary Specialists and Animal Scientists (AMEVEA—Asociación Colombiana de Veterinarios y Zootecnistas Especialistas en Avicultura) as the primary sampling frame. AMEVEA was selected because it represents the leading technical association of poultry practitioners in Colombia, providing access to a qualified target population. Given the lack of a centralized national registry of all poultry professionals, this combined sampling approach offered a pragmatic method for participant recruitment. Inclusion criteria for respondents were as follows: (a) hold a professional degree from a Colombian university and (b) be actively working in the poultry sector with a minimum of 1 year of professional experience.

### 2.3. Survey Structure

The survey comprised 23 statements and 3 multiple‐choice questions distributed across seven sections (for the complete survey instrument, see Supporting Information [available here]). Section A, the introductory section, provided information for participants, including the study objectives, the institutional affiliation of the research team, its noncommercial research purpose, and details regarding data confidentiality and voluntary participation, the estimated completion time, and the informed consent declaration. Section B collected sociodemographic data (gender, age, and undergraduate and postgraduate educational background). Section C gathered information on professional and occupational characteristics (years of professional experience, type of production system, prior training in AW, and the institution providing such training). Sections D, E, and F assessed, respectively, self‐perceived knowledge/familiarity (6 statements), attitudes (8 statements), and perceptions (9 statements) of professionals regarding AW. Responses across these three sections were recorded using a five‐point Likert scale, ranging from 1 (“*strongly disagree*”) to 5 (“*strongly agree*”) [11]. Given that the statements in Section D consisted of technical propositions regarding AW principles, respondents’ degree of agreement served as a proxy for their self‐perceived knowledge and conceptual alignment with these topics, an approach previously utilized in similar veterinary survey‐based research [28]. However, this measure reflects self‐perceived alignment and subjective understanding rather than an objective test of technical knowledge. Finally, Section G assessed the barriers, opportunities, and limitations perceived by respondents in the implementation of AW standards on broiler and laying hen farms. For each of these three dimensions, respondents were required to select exactly three statements from a predefined list of eight options, identifying those they considered most relevant to their professional context. The internal reliability of the instrument was assessed using Cronbach’s alpha coefficient for each subscale. The values obtained were *α* = 0.70 for the knowledge subscale (6 items), *α* = 0.80 for the attitude subscale (8 items), and *α* = 0.80 for the perception subscale (9 items), indicating good internal consistency in accordance with the criteria established by George and Mallery [29]. The complete survey instrument used to collect demographic, perception, and attitude data is available in Appendix A1.

### 2.4. Data Analysis

Stata Version 19.0 (College Station, TX, USA) software was used for all statistical analyses. A descriptive analysis was performed on the information obtained from the 106 completed surveys. A probability level of *p* < 0.05 was chosen as the limit for statistical significance in all tests. For each thematic section (knowledge, attitudes, and perceptions), a total score was calculated using the simple summation of the corresponding Likert scale items, and descriptive analyses of these variables were subsequently performed. The construction of professional profiles was developed through an integrated hierarchical classification strategy implemented in two phases [30, 31]. Profile determination was achieved by combining the visual inspection of the dendrogram with the quantitative analysis of the agglomeration schedule (Supporting Table S1). Truncation was set at $*k* = 4$ based on the sharp increase in the agglomeration coefficient when reducing to three clusters, confirming four groups as the most parsimonious and distinct configuration.

In the first phase, three independent factor analyses were applied according to the nature of each variable block. For the total scores of knowledge, attitudes, and perceptions (variables of a quantitative nature), a principal component analysis (PCA) was performed. Prior to analysis, the underlying assumptions were verified using Bartlett’s test of sphericity ($\chi^2 = 132$, $df = 3$, $*p* < 0.001$) and the Kaiser–Meyer–Olkin measure of sampling adequacy (overall KMO = 0.629), confirming the suitability of the data for dimensionality reduction [32]. The number of components to retain was determined through parallel analysis [33], yielding a single component that explained 72.93% of the total variance, with factor loadings of 0.753 (knowledge), 0.923 (attitudes), and 0.878 (perceptions). Two independent multiple correspondence analyses (MCAs) were subsequently performed: one for the categorical sociodemographic variables and one for the perceived barriers, opportunities, and limitations. The selection of dimensions to retain in each MCA was based on the cumulative inertia criterion and the principle of parsimony. The first nine dimensions were retained, accounting for 64.6% of the total inertia, a threshold considered adequate for multiple categorical variables in health sciences [30, 34] while ensuring the conceptual interpretability and coherence of the resulting profiles [35].

In the second phase, the individual factor coordinates obtained from the individual PCA and MCA analyses were concatenated into an integrated matrix using multiple factor analysis (MFA) principles, where coordinates were weighted by the first eigenvalue of each individual analysis to balance the contribution of quantitative and qualitative data blocks. A single hierarchical cluster analysis using Ward’s method was then applied to this matrix, grouping individuals by minimizing within‐cluster variance [35]. This procedure yielded four professional profiles that comprehensively synthesize the heterogeneity of professionals across all evaluated dimensions: sociodemographic characteristics, knowledge, attitudes, perceptions, and perceived barriers, opportunities, and limitations regarding AW in the Colombian commercial poultry industry. The characterization of each profile was conducted through the analysis of the modalities with the greatest statistical weight, identified using the *v*‐test, with those reaching $*p* < 0.05$ considered statistically significant [30]. The multiple‐choice responses from the barriers, limitations, and opportunities section were analyzed using absolute frequencies and percentages.

## 3. Results

### 3.1. Sociodemographic and Employment Characteristics

The survey was completed by 106 professionals affiliated with the Colombian commercial poultry industry, of whom 58 were male (54.7%) and 48 were female (45.3%). The majority of respondents were between 38 and 57 years of age (*n* = 60; 56.6%), with professional experience ranging from one to 49 years (mean 17.2 ± 11.3 years) (Table 1). No statistically significant differences were observed in the distribution by production system or postgraduate educational background (*p* > 0.05). A total of 70.8% of professionals had received prior training in AW, of whom 55.7% had been trained by public institutions (*n* = 59)—including the Instituto Colombiano Agropecuario (ICA), the Instituto Nacional de Vigilancia de Medicamentos y Alimentos (INVIMA), and several public universities in Colombia—9.4% (*n* = 8) by private entities such as the Federación Nacional de Avicultores de Colombia (FENAVI), and 5.7% (*n* = 6) by both public and private institutions.

**TABLE 1:** Sociodemographic and occupational characteristics of poultry professionals (*n* = 106).

Variable	Category	*n* (%)
Age	18–27	6 (5.7)
	28–37	21 (19.8)
	38–47	27 (25.5)
	48–57	33 (31.1)
	≥ 58	19 (17.9)

Experience	0–10	32 (30.2)
	11–20	36 (34.0)
	21–30	25 (23.5)
	31–49	13 (12.3)

Undergraduate	Veterinary medicine	22 (20.8)
	Animal scientist	65 (61.3)
	Veterinarian medicine and animal scientist	19 (17.9)

Postgraduate level	None	42 (39.6)
	Specialization	36 (34.0)
	Master’s degree	22 (20.8)
	Doctorate	6 (5.7)

Type of production	Egg‐laying bird	34 (32.1)
	Broiler chicken	33 (31.1)
	Both (layer and broiler)	39 (36.8)

### 3.2. Assessment of the Levels of Knowledge, Attitude, and Perception

Respondents demonstrated a heterogeneous level of knowledge regarding AW. Considering the proportion of respondents who selected Likert scale values 4 and 5, a high level of agreement was observed in the recognition of the impact of stress on poultry product quality, including eggs and meat (95.3%), as well as in the importance of having personnel trained in AW (93.4%), a finding that suggests a positive valuation of technical training in this domain. In the same direction, although at a lower proportion (78.3%), respondents agreed on the existence of a relationship between animal‐based indicators and on‐farm environmental conditions, as reflected in the items concerning the presence of pododermatitis and keel bone integrity, and plumage soiling.

However, greater divergence was observed in knowledge responses. Regarding the conceptual definition of AW, only 41.5% of respondents identified the option aligned with scientifically established international standards. Concerning the management of birds exhibiting severe feather pecking, 68.9% considered that affected individuals should be subjected to euthanasia, while the remaining proportion reported uncertainty regarding the criteria for making this decision at the farm level. A similar pattern of uncertainty and divergent opinions was observed regarding beak management: A significant proportion of respondents agreed that beak trimming should be performed indiscriminately across all poultry production systems (Figure 1).

**FIGURE 1:**
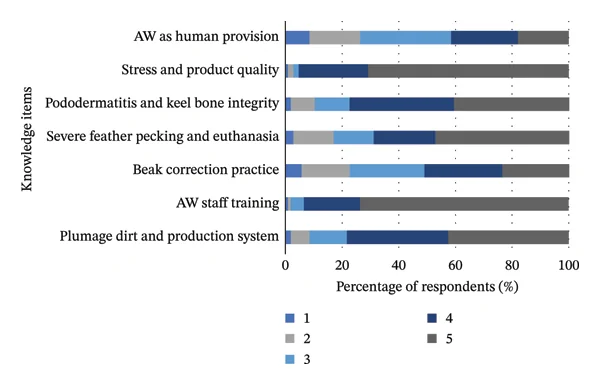
Distribution of responses to knowledge items on a 5‐point Likert scale (1 = *strongly disagree* to 5 = *strongly agree*). The horizontal axis shows the percentage of respondents (%). AW = animal welfare. Item labels are abbreviated for display purposes; full statements are provided in the Supporting Information (available here).

Overall, the attitudes of professionals toward poultry AW were predominantly favorable (Figure 2). Regarding core biological and ethical principles, high agreement was observed for the recognition of the capacity of birds to experience pain (93.4%); the necessity of expressing natural behaviors (90.6%); the premise that poultry welfare is as important as human welfare (90.6%); the acknowledgment of avian preferences, motivations, and aversions (89.6%); and the principle that birds should be free from fear and distress (85.8%).

**FIGURE 2:**
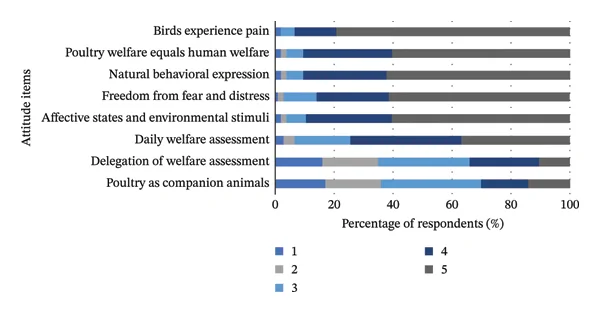
Distribution of responses to attitude items (Likert scale: 1 = *strongly disagree*, 2 = *disagree*, 3 = *neither agree nor disagree*, 4 = *agree*, 5 = *strongly agree*). The horizontal axis shows the percentage of respondents (%). AW, animal welfare. Item labels are abbreviated for display purposes; full statements are provided in the Supporting Information (available here).

In contrast, lower agreement was reported for operational and management items. While 74.5% supported daily AW assessment and recording, only 34.0% agreed with delegating this task to external personnel. Similarly, a low level of agreement (30.2%) was observed regarding the potential consideration of commercial poultry as companion animals, reflecting a clear distinction between farm AW and pet status.

Professionals’ perceptions regarding AW implementation in the commercial poultry industry were predominantly positive concerning the relevance, impact, and assessment criteria of welfare standards (Figure 3).

**FIGURE 3:**
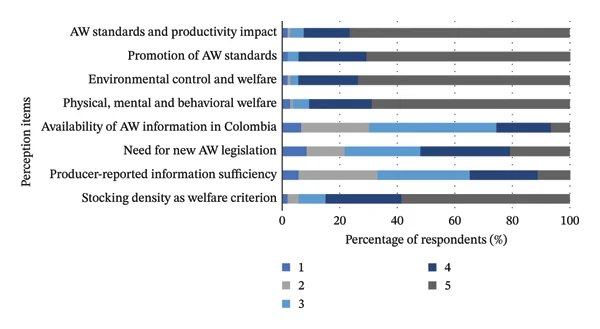
Distribution of responses to perception items (Likert scale: 1 = *strongly disagree*, 2 = *disagree*, 3 = *neither agree nor disagree*, 4 = *agree*, 5 = *strongly agree*). The horizontal axis shows the percentage of respondents (%). AW, animal welfare. Item labels are abbreviated for display purposes; full statements are provided in the Supporting Information (available here).

A high proportion of respondents agreed or strongly agreed that promoting AW standards is appropriate and beneficial (94.4%), and that applying these standards positively impacts bird health and industry productivity (92.4%). Regarding technical criteria, strong support was observed for considering environmental regulation—such as temperature, humidity, and ventilation—as a guarantee of welfare (94.4%), evaluating physical health, mental state, and natural behavior as indispensable components (90.6%), and incorporating stocking density (in litter or cage systems) within evaluation frameworks (84.9%).

Conversely, a more critical perception emerged regarding information transparency and regulatory frameworks in the local context. Only 25.5% of respondents considered that sufficient information on poultry AW is available in Colombia, and only 34.9% perceived that information provided by producers is sufficient to evaluate on‐farm conditions. Consequently, 51.9% agreed that new legislation is required to prevent suffering and ensure adequate management standards in the sector.

### 3.3. Assessment of Barriers, Opportunities for Improvement, and Limitations

Regarding the factors influencing AW implementation in the commercial poultry sector (Table 2), findings were categorized into perceived barriers, technical knowledge gaps, and opportunities for development.

**TABLE 2:** Frequencies and percentages of responses regarding barriers, limitations, and opportunities for improvement in animal welfare according to poultry professionals (*n* = 106).

Variable/item	Yes	No
	*n* (%)	*n* (%)
*Perceived Industry Barriers in Colombia*		
Difficult access to training for professionals.	18 (17.0)	88 (83.0)
Lack of knowledge on the part of professionals.	43 (40.5)	63 (59.5)
The lack of motivation for economic investment on the part of the producers.	60 (56.6)	46 (43.4)
The lack of sufficient incentives for producers.	38 (35.8)	68 (64.2)
Beliefs about declining productivity and economic gains.	47 (44.3)	59 (55.7)
The difficulty in arranging and planning visits to poultry farms.	9 (8.5)	97 (91.5)
Resistance to change.	50 (47.2)	56 (52.8)
The impact on profitability due to the decrease in density in the sheds.	41 (38.7)	65 (61.3)

*Factors Improving Adoption and Willingness (Opportunities)*		
The creation of economic incentives for producers.	55 (51.9)	51 (48.1)
The willingness of certain consumers to pay more for meat and eggs that meet animal welfare standards.	51 (48.1)	55 (51.9)
The ease of access to training for producers.	28 (26.4)	78 (73.6)
Evidence on the relationship between the implementation of animal welfare practices and economic gains.	65 (61.3)	41 (38.7)
The best training for professionals.	20 (18.9)	86 (81.1)
Timely, frequent, and conscious visits by professionals to production units, with animal welfare criteria.	28 (26.4)	78 (73.6)
Assertive and proactive communication by professionals regarding their concerns about animal welfare factors.	31 (29.2)	75 (70.8)
The conviction on the part of producers regarding the need for professionals to have the knowledge and ability to implement animal welfare.	29 (27.4)	77 (72.6)

*Limitations Faced When Promoting Standards with Producers*		
Personal lack of knowledge of the standards and their application.	44 (41.5)	62 (58.5)
The limited ability to influence, as a professional, the decisions of the producers.	33 (31.1)	73 (68.9)
Deeply rooted practices among producers prevent substantial changes on this issue.	62 (58.5)	44 (41.5)
The producer’s lack of confidence in the professional’s knowledge.	19 (17.9)	87 (82.1)
Deeply rooted practices in professional practice that prevent a change in attitude.	27 (25.5)	79 (74.5)
The producers’ rejection of this issue stems from their view that the change in the number of animals per production area is negative.	54 (50.9)	52 (49.1)
The lack of communication and trust between the professionals and the producer.	14 (13.2)	92 (86.8)
The perception of animal welfare as something that is not essential in production.	51 (48.1)	55 (51.9)

The main perceived structural and economic barriers reported by respondents were producers’ lack of motivation for financial investment (56.6%), general resistance to change (47.2%), and concerns regarding potential declines in productivity and economic returns (44.3%). At the individual level, a critical knowledge gap was identified: A significant proportion of professionals cited a personal lack of familiarity with existing AW standards as a primary operational limitation.

Conversely, key opportunities for improvement focused heavily on economic drivers and capacity building. A majority of professionals (61.3%) emphasized the need to generate solid evidence demonstrating the link between AW practices and financial performance. Additionally, respondents highlighted the creation of economic incentives for producers (51.9%) and growing consumer willingness to pay a price premium for welfare‐certified poultry products (48.1%) as viable pathways to facilitate industry adoption.

### 3.4. Professional Profiles

The professional typology analysis identified four distinct clusters differentiated by sociodemographic variables, knowledge, attitudes, and perceptions, as well as by the assessment of barriers, limitations, and opportunities for the implementation of AW standards in the Colombian commercial poultry industry. The classification was obtained through hierarchical cluster analysis using Ward’s method applied to the factor coordinates of the first nine MCA dimensions, which accounted for 64.6% of the total inertia.

To maintain analytical rigor, cluster characterization was strictly grounded in variables displaying statistically significant differences based on *v*‐test values (*v*‐test > 1.96, *p* < 0.05). The titles assigned to each profile represent interpretative labels proposed by the authors to aid conceptual synthesis rather than empirical parameters.

#### 3.4.1. Profile 1. Technical Welfare Leaders (*n* = 27; 25.5%)

A higher proportion of respondents with advanced academic training and strong technical familiarity with AW concepts characterized this profile. Members expressed significant agreement regarding the necessity of natural behavioral expression in birds (*v*‐test = 2.39; *p* = 0.017) and stocking density as an AW indicator (*v*‐test = 2.35; *p* = 0.019). Furthermore, these professionals significantly rejected the belief that AW reduces productivity, as evidenced by their nonselection of reduced profitability due to decreased density as a relevant barrier (*v*‐test = 3.50; *p* < 0.001). The main opportunities prioritized by this group were increased professional training (*v*‐test = 4.13; *p* < 0.001) and improved access to training for producers (*v*‐test = 3.27; *p* = 0.001). From a conceptual perspective, this group displays characteristics associated with technical expertise and educational leadership within the sector.

#### 3.4.2. Profile 2. Convinced Professionals With Structural Barriers (*n* = 37; 34.9%)

This cluster exhibited favorable attitudes toward AW and identified assertive communication and technical farm visits as opportunities for improvement. However, respondents in this group predominantly selected items reflecting external and operational constraints, specifically identifying the perception that AW is nonessential to production as a significant limitation (*v*‐test = 3.72; *p* < 0.001). Notably, the lack of producer trust in professionals’ knowledge was significantly unselected (*v*‐test = 3.88; *p* < 0.001), indicating that obstacles reported by this profile pertain primarily to organizational and structural farm factors rather than interpersonal trust.

#### 3.4.3. Profile 3. Professionals With Relational Barriers (*n* = 16; 15.1%)

Composed significantly of older professionals (*v*‐test = 3.17; *p* = 0.002) with lower rates of postgraduate education (*v*‐test = 2.48; *p* = 0.013), this profile was distinguished by relational challenges. Its members significantly identified producers’ lack of trust in professionals’ knowledge (*v*‐test = 5.53; *p* < 0.001) and deficient communication between professionals and producers (*v*‐test = 4.56; *p* < 0.001) as the primary limitations. Conversely, general producer resistance to change was significantly less selected as a barrier by this group (*v*‐test = 2.45; *p* = 0.014), highlighting a profile primarily focused on interpersonal and trust‐building dynamics between technical advisors and farmers.

#### 3.4.4. Profile 4. Results‐Oriented Pragmatists (*n* = 26; 24.5%)

This profile was characterized by greater professional experience (> 20 years; *v*‐test = 2.69; *p* = 0.007) and a higher rate of prior AW training (*v*‐test = 2.32; *p* = 0.020). Members prioritized economic and operational feasibility, identifying evidence linking AW to financial returns as a primary opportunity (*v*‐test = 3.36; *p* < 0.001) and assertive communication as a key coordination mechanism (*v*‐test = 3.47; *p* < 0.001). Items regarding lack of technical conviction or training needs were not significantly selected, indicating a profile oriented toward practical efficiency and economic justification.

## 4. Discussion

The present study constitutes one of the first comprehensive analyses of knowledge, attitudes, perceptions, barriers, opportunities for improvement, and limitations regarding AW among professionals affiliated with the commercial poultry industry in Colombia. The results demonstrate a high overall level of appropriation of the AW construct, accompanied by specific conceptual gaps and marked heterogeneity in professional profiles, with relevant implications for the formulation of sector‐level policies and differentiated intervention strategies.

### 4.1. Knowledge, Attitudes, and Perceptions of Professionals Regarding AW

The results of the present study indicate that professionals affiliated with the Colombian commercial poultry industry exhibit predominantly favorable attitudes toward AW and a solid recognition of its productive implications, yet with relevant conceptual gaps and divergences in clinical management criteria. This pattern is consistent with findings reported in Latin American and European contexts, where veterinary professionals demonstrate positive attitudes toward AW but encounter limitations in its practical application [12, 36].

The conceptual ambiguity detected in the definition of AW, whereby 41.5% of respondents agreed that AW encompasses everything that humans provide to animals, reveals a cross‐cutting weakness in the understanding of the construct that transcends the level of technical knowledge. This finding is consistent with recent literature, indicating that, although the concept of AW has evolved toward multidimensional frameworks such as the five domains model [37], its practical internalization tends to focus on physical indicators rather than affective or cognitive dimensions [38]. The updated version of this model emphasizes precisely the centrality of positive and negative affective states as the core of welfare, requiring a level of understanding that transcends the mere absence of disease or the provision of basic material conditions [38].

Indeed, the near‐unanimous recognition of technical indicators such as the association between pododermatitis and environmental quality (78.3%), the relationship between plumage soiling and management conditions (78.3%), and the need for personnel training (93.4%) contrasts with the limited conceptual integration of welfare as a state involving subjective experiences, mental states, and motivational processes. International evidence underscores that modern AW assessment in intensive systems requires the integration of animal‐, environment‐, and management‐based indicators alongside emotional state metrics [38, 39]. In this regard, the near‐automatic association between the control of environmental parameters, such as temperature, humidity, and ventilation, and the guarantee of welfare, endorsed by 94.4% of respondents, may be interpreted as a technocentric view of AW centered on management variables. Although these parameters are essential, the literature cautions that welfare cannot be reduced to isolated environmental variables but requires multifactorial, animal‐centered assessment [27]. In this context, the limited conceptual appropriation observed suggests the need to strengthen both the conceptual and ethical foundations of AW, even among professionals with high technical performance.

The high level of agreement regarding the impact of stress on product quality (95.3%) reflects that professionals recognize AW as a relevant productive factor rather than as an isolated ethical imperative. This instrumental approach to AW, oriented toward economic and sanitary outcomes, has been documented as an effective strategy for the adoption of welfare practices in intensive systems [22, 40] and could represent a strategic entry point for the design of training interventions in the Colombian context. However, reducing AW exclusively to its productive dimension carries the risk of overlooking ethical and affective aspects that are central to current international regulatory frameworks [41].

The attitudes of professionals were predominantly favorable, with the recognition of birds’ capacity to experience pain representing the aspect with the highest level of consensus (93.4%). This finding is relevant and aligns with the growing body of scientific evidence supporting the capacity of birds to experience pain and complex affective states [42]. Nevertheless, the existence of professionals who deny this capacity constitutes a critical concern, given that the acceptance of sentience is both an ethical and technical prerequisite for the coherent implementation of AW standards [43]. The near‐universal acceptance of sentience in terms of pain coexists with greater dispersion in the recognition of more complex dimensions, such as fear and distress (85.8% agreement), preferences and motivations (89.6%), and natural behavioral expression (90.6%), suggesting a gradient understanding of the affective life of birds that diminishes as it moves away from the most visible indicators most directly linked to productive management.

The tendency toward neutrality and disagreement regarding the possibility of considering hens or broiler chickens as companion animals, with 35.9% disagreeing and 34.0% remaining neutral, illustrates how the act of categorizing an animal as food plays a determining role in the moral value attributed to it [44]. This phenomenon, known as the meat paradox or the moral categorization effect, implies that different moral value is attributed to different animal species even when their sentience is recognized [45]. In the context of commercial poultry production, this categorization may function as an implicit barrier to the adoption of more demanding AW standards, by reducing the perceived moral relevance of bird suffering in intensive systems.

The main differences in attitudes are expressed in the incorporation of the emotional component and in the valuation of systematic assessment using formal instruments. Daily AW assessment was endorsed by 74.5% of professionals; however, 18.9% remained neutral, indicating that not all respondents consider this practice to be a priority or feasible within their professional activities. This is consistent with recent studies indicating that the effective adoption of welfare standards depends not only on knowledge but also on the internalization of values and the perception of professional responsibility [8]. The considerable proportion of professionals who consider AW assessment to be delegable to external consultants (34.0% agreement) suggests potential tensions between internal technical responsibility and the externalization of quality control—a phenomenon documented in productive systems where certification and auditing tend to replace internal processes of continuous improvement [46].

The perceptions of professionals were predominantly positive regarding the impact and relevance of AW standards, but markedly critical with respect to the availability of information in the Colombian context. The fact that only 25.5% of respondents considered that sufficient information on AW in the Colombian poultry industry is available, and that 34.9% estimated that information provided by producers is sufficient to determine AW conditions, which reflects a regulatory environment perceived as incomplete or poorly coordinated. At the international level, regulatory frameworks have evolved toward increasingly demanding standards, driven by both social pressure and market requirements [47]. In Latin America, however, implementation remains heterogeneous and is frequently subject to debate among the various stakeholders involved [48].

The tendency of 51.0% of professionals to agree with beak correction without exception was calculated by combining responses “*Agree*” [4] and “*Strongly Agree*” [5] on the 5‐point Likert scale for the item evaluating mandatory beak trimming warrants particular attention, given that in Colombia, this practice is subject to precise specifications under current regulations [6]. This regulatory knowledge gap, which coexists with generally high levels of technical knowledge, suggests that the deficit is not one of general information but rather of articulation between professional training and the specific national regulatory framework. This disconnect between technical knowledge and regulatory knowledge has been identified as a frequent obstacle to the implementation of AW standards in contexts where regulation is relatively recent [14, 47]. Similarly, the divided positions on the need for new legislation (51.9% in agreement versus 21.7% in disagreement) reflect heterogeneous perceptions regarding the sufficiency of the current regulatory framework, underscoring the importance of targeted communication and regulatory dissemination strategies directed specifically at veterinary professionals working in the poultry sector.

### 4.2. Barriers, Opportunities for Improvement, and Limitations Perceived by Professionals Regarding AW

The main barriers identified lack of economic motivation among producers, resistance to change, and beliefs regarding reduced profitability confirm that the obstacles are predominantly structural and cultural rather than cognitive. This finding is consistent with recent studies, indicating that the perception of negative economic impact constitutes one of the main constraints to the adoption of welfare practices in intensive production systems [46, 49]. Paradoxically, most professionals recognize the existing evidence supporting the relationship between AW and economic gains as an opportunity, which is consistent with findings demonstrating that improvements in welfare may be associated with better productive indicators and reduced losses [10, 50]. This tension suggests that the barrier is not the absence of evidence, but rather its effective translation into production‐related decision‐making. Opportunities for improving the implementation of AW standards were all related to economic incentives directed toward producers, including consumers’ willingness to pay more for products produced under AW conditions, which aligns with global trends in which consumers shape product pricing [51, 52].

The identification of deeply rooted practices and the perception of AW as a nonessential component of production by producers, as the main limitations, reinforces the cultural dimension of the problem and is consistent with findings reported in other studies [13, 53]. The literature on organizational change in agricultural systems indicates that the transformation of traditional practices requires interventions combining economic incentives, technical training, and sectoral leadership [54]. Finally, professionals acknowledge their own lack of knowledge regarding AW standards and their implementation; this may be related to limited interest in and exposure to poultry production and AW during university training [55, 56], indirectly suggesting gaps in applied knowledge related to poultry welfare.

### 4.3. Professional Profiles

The similarity in knowledge, attitudes, and perceptions scores among the four groups, with differences of less than two points in the means, indicates that heterogeneity among professionals does not lie in the level of knowledge or in the direction of their attitudes, but rather in the way they perceive and value structural conditions such as economic incentives for the implementation of AW. This finding is relevant because it challenges the effectiveness of uniform educational interventions as the sole improvement strategy and points to the need for differentiated approaches according to professional profile [22, 36].

Profile 1, “Technical leaders in welfare” (25.5%), is consistent with findings reported in the context of bovine veterinary medicine, where professionals with a stronger emphasis on working directly with animals exhibited more favorable attitudes toward the active promotion of positive welfare, beyond merely preventing negative experiences [57]. The existence of this profile within the Colombian poultry sector suggests the presence of a professional core with genuine technical leadership capacity, from which interventions may be directed toward groups with lower levels of appropriation. However, professionals with high awareness of welfare themselves acknowledge structural limitations of their role, noting that final decisions regarding animal management ultimately rest with producers as owners, thereby limiting their capacity for direct influence [58].

Profile 2, “Convinced but facing structural barriers” (34.9%), illustrates a tension widely documented in the literature: the coexistence of favorable attitudes toward AW with a perceived low capacity to influence producers’ decisions. Members of this group perceive AW as a nonessential component of production as the predominant limitation, while lack of trust between the producer and the professional does not constitute a relevant obstacle, since none of the profile members selected this option. This suggests that the perceived barrier is structural and organizational rather than interpersonal in nature. Relational factors such as mutual trust, shared time, and the ability to sustain cooperative discussions have already been documented as determinants for the effective adoption of changes related to prevention and implementation of standards [58]. The absence or weakness of these relational factors, rather than technical ignorance, may explain the gap between conviction and practical action characterizing this profile. Collaborative applied educational interventions, in which veterinarians and producers share joint learning spaces, have significant potential to foster partnerships capable of addressing communication and profitability barriers by demonstrating, through data, the actual cost–benefit relationship of preventive management [14].

Profile 3, “Professionals facing relational barriers” (15.1%), presents characteristics warranting particular attention. Its composition, predominantly consisting of older professionals with lower levels of postgraduate education, is consistent with findings from studies involving poultry producers in contexts where declared knowledge coexists with practical resistance to change, responding to an entrenched productivist logic that requires interventions extending beyond conventional technical training [59]. The predominant limitations identified in this profile, namely, producers’ lack of trust in the professional’s knowledge and poor communication between professionals and producers, indicate that relational difficulties constitute the main perceived obstacle. Importantly, the association between older age and lower postgraduate education with this specific profile does not imply that sociodemographic variables are general predictors of AW appropriation within the overall sample, given that Profiles 2 and 4 showed no statistically significant sociodemographic differentiation. Rather, the data suggest that, within this particular subgroup, professional trajectory and level of academic updating interact with specific relational difficulties, shaping a differentiated pattern of perceived barriers.

Profile 4, “Results‐oriented pragmatists” (24.5%), presents the highest level of professional experience and the highest rate of prior AW training, suggesting that its orientation toward the economic benefits of AW is not the result of ignorance, but rather of a deliberate and consolidated position. This profile reflects a pattern consistently documented in intensive production systems, where producers consider the perception of concrete monetary or nonmonetary benefits essential for adopting welfare‐friendly practices, since improvements in animal health may translate into greater productivity, improved feed efficiency, and reduced veterinary costs [60]. For this profile, the most effective strategy is not ethical sensitization, but rather contextualized empirical demonstration of the economic return of AW. Research in poultry systems has documented that the appropriate implementation of welfare principles effectively reduces losses resulting from carcass condemnations due to lesions, thereby contributing to increased profitability, although this level of evidence has not yet become readily accessible to all stakeholders within the Latin American sector [61].

From a strategic perspective, the results indicate that interventions should be differentiated and adapted to each profile. Studies have shown that the identification of different behavioral types within the production sector may help reduce disease risk [62] and improve the adoption of practices within production systems [63, 64]. For Profile 1, strengthening governance instruments and technical leadership mechanisms is required to enhance their influence capacity, whereas Profile 2 requires interventions focused on communication and negotiation skills with producers; Profile 3 requires intensive updating and sensitization programs simultaneously addressing educational gaps and relational difficulties; and Profile 4 requires economic and demonstrative strategies supported by contextualized evidence regarding the return on investment of AW within the Colombian poultry sector.

Taken together, the four identified profiles underscore that the heterogeneity of the veterinary professional field in Colombian poultry production responds to complex processes involving organizational, relational, and professional trajectory‐related factors, beyond fixed individual characteristics. The results are consistent with the literature documenting the complexity of AW practice adoption processes in intensive systems, where relational, economic, and institutional factors interact with professionals’ individual characteristics to determine implementation patterns [12, 36, 58].

### 4.4. Limitations of the Study

The limitations of the study are related to: (a) the statistical representativeness of the results and the inference of these findings to the overall population of professionals involved in Colombian commercial poultry production, because convenience sampling was used; therefore, the results should be interpreted as exploratory and their generalization should be approached with caution; (b) potential information bias, because professionals who voluntarily agreed to participate in the study may have had greater prior interest in or awareness of AW, which may have led them to respond more frequently, potentially generating an overestimation of knowledge levels and positive attitudes; (c) the use of a nonprobabilistic sampling strategy combining convenience and snowball sampling introduces centered on AMEVEA members introduces potential selection bias. While AMEVEA is a primary benchmark in the sector, professionals not affiliated with formal networks may be underrepresented, so caution is warranted when generalizing these results to the entire population of Colombian poultry practitioners. Professionals who opted to participate may have possessed greater interest, digital literacy, or motivation regarding poultry health management compared to nonrespondents. This self‐selection dynamic may have influenced the profile, relative proportions, and key characteristics of the four identified clusters. Consequently, while these clusters offer valuable insights into the diversity of practices and perspectives within the surveyed cohort, the findings should be interpreted as reflective of this specific sample rather than as fully representative parameters of the entire Colombian poultry industry. (d) Knowledge regarding AW was assessed based on agreement with technical statements (self‐perceived knowledge) rather than through an objective examination of technical skills or factual accuracy. (e) Limited professional coverage, because the study was primarily directed toward professionals affiliated with a sectoral association and with access to digital platforms, which may have excluded professionals from rural areas or with lower connectivity; (e) the cross‐sectional rather than longitudinal design of the study, which provides a “snapshot” of professional perceptions at a specific point in time; and (f) the results are based exclusively on self‐reported statements by the professionals themselves. These last two aspects limit the possibility of establishing causal relationships and evaluating changes over time. It is necessary to conduct longitudinal studies evaluating the association and correspondence between the attitudes and knowledge reported by professionals through surveys and actual practices under field conditions, using studies that assess practices through direct observation or farm audits. Such approaches would provide a more valid basis for guiding training programs and improvement strategies and, additionally, for determining whether training interventions effectively modify the identified professional profiles.

## 5. Conclusion

Professionals involved in Colombian commercial poultry production exhibited predominantly favorable attitudes toward AW, with a high level of recognition of bird sentience (93.4%), the importance of environmental control (94.4%), and the impact of production‐related stress on product quality (95.3%). However, relevant conceptual gaps were identified: Only 41.5% agreed with the comprehensive definition of AW, and 51% agreed with beak trimming without exception, a practice whose application is conditional under current Colombian regulations. These gaps coexist with acceptable levels of technical knowledge, indicating that the limitation lies not in general information, but rather in conceptual appropriation and articulation with the national regulatory framework.

The main perceived barriers to the implementation of AW standards were producers’ lack of motivation for economic investment (56.6%), resistance to change (47.2%), and beliefs regarding decreased productivity and profits (44.3%). The most frequently identified opportunities for improvement were evidence regarding the relationship between AW and economic gains (61.3%), the creation of economic incentives for producers (51.9%), and consumers’ willingness to pay more for products produced under AW standards (48.1%). The predominant limitations were producers’ deeply rooted practices (58.5%), the perception of AW as a nonessential component of production (48.1%), and professionals’ own lack of knowledge regarding standards and their implementation (41.5%). Taken together, these findings indicate that the barriers and limitations to AW implementation are located predominantly at the producer level and within the organizational context of the sector, rather than at the level of professional knowledge or attitudes.

The integrated hierarchical cluster analysis performed on the factorial coordinates derived from the PCA and the two MCA analyses enabled the construction of four statistically differentiated professional profiles through *v*‐test analysis (*p* < 0.05): Technical leaders in welfare (25.5%), Convinced but facing structural barriers (34.9%), Professionals facing relational barriers (15.1%), and Results‐oriented pragmatists (24.5%). The similarity in knowledge, attitudes, and perceptions scores among the four profiles, with mean differences of less than two points, indicates that heterogeneity among professionals is not determined by level of knowledge or direction of attitudes, but rather by the way each group perceives and values the barriers, opportunities, and limitations associated with AW implementation. In response to the general objective of the study, the comprehensive characterization of professionals involved in Colombian commercial poultry production demonstrates that these professionals possess a favorable attitudinal and technical foundation toward AW; however, heterogeneity among profiles—particularly regarding the perception of structural, relational, and economic barriers—constitutes the principal differentiating factor between groups and the key element that should guide the design of differentiated intervention strategies for the effective implementation of AW standards within the Colombian poultry sector.

## Author Contributions

Conceptualization: Marlyn H. Romero and Laura García‐Naranjo; methodology: Marlyn H. Romero and Laura García‐Naranjo; data collection: Laura García‐Naranjo; analysis: Laura García‐Naranjo and Marlyn H. Romero; writing–original draft preparation: Laura García‐Naranjo and Marlyn H. Romero; writing–review and editing: Marlyn H. Romero and Laura García‐Naranjo; writing–final draft preparation: Marlyn H. Romero and Laura García‐Naranjo; funding acquisition: Marlyn H. Romero.

## Funding

This project was funded by the Research Investigations and Vice Chancellor of the University of Caldas.

## Conflicts of Interest

The authors declare no conflicts of interest.

## Supporting Information

Additional supporting information can be found online in the Supporting Information section.

## Supporting information


**Supporting Information** Supporting Table S1: Quantitative validation of cluster truncation (*k* = 2–5) including agglomeration schedule metrics and Ward’s within‐cluster inertia jumps. Appendix A1: Complete assessment instrument (Survey Questionnaire) used to evaluate animal scientists’ and veterinarians’ knowledge, attitudes, perceptions, barriers, limitations, and opportunities regarding poultry animal welfare in Colombia.

## Data Availability

The data used to support the findings of this study are available from the corresponding author upon request.
