# Supplementary material for: Animal Welfare in Colombian Poultry Industry: Exploring Veterinarians’ Perceptions, Attitudes, Knowledge, Barriers, and Professional Profiles
Source: Vet Med Int. 2026 Oct 6;2026:3976972. doi: 10.1155/vmi/3976972 (PMC13640050; doi:10.1155/vmi/3976972)
Supplement: Supplementary file 1 [file VMI-2026-3976972-s001.zip › 3976972-Supplementary S1.docx]

**S1. Survey Questionnaire**

**Animal Welfare in Colombian poultry industry: Exploring veterinarians' perceptions, attitudes, knowledge, barriers and professional profiles.**

Greetings !

The purpose of this survey is to recognize some basic aspects of the perceptions, knowledge, attitudes and opportunities for improvement of veterinarians and/or animal scientists in the application of animal welfare standards in the Colombian poultry industry.

The data obtained will be anonymous and its use will be exclusively for academic purposes, therefore, the information will not be subject to public, open or general disclosure.

The survey is divided into six components that have an estimated response time of 15 minutes.

We appreciate your collaboration, with these answers that contribute to expanding knowledge about animal welfare in the poultry industry in Colombia.

**Component one: identification data**

*Select the option or complete the corresponding information:*

1. Select your gender identity

- Female
- Male
- Other

2. Place your age within one of the following ranges:

- Between 18 and 27 years old
- Between 28 and 37 years old
- Between 38 and 47 years old
- Between 48 and 57 years old
- 58 years or older.

Consider whether to leave it as is, given the sensitivity of the question. There's a chance they might lie or not answer.

3. What is your undergraduate background?

- Veterinarian
- Animal scientist
- Veterinarian and animal scientist
- Agricultural administrator
- Agricultural Engineer
- Food Engineer
- Another, which____________________________________

4. Do you have postgraduate training?

- Yeah
- No

5. If your answer is yes, please select:

- Specialization
- Mastery
- Doctorate

**Component two: general data**

6. How many years of experience do you have working in the poultry sector? ___
7. In which department(s) of Colombia do you work? ___________________
8. What type of poultry production do you dedicate most of your professional practice to?

- Laying hens
- Broiler chicken
- Both
- Another, which one? ________________________________________________

9. What is the minimum and maximum number of birds allowed on the farms you advise?

Max____ Min____

10. Have you received formal training in animal welfare?

- Yeah
- No

11. If your answer is yes, please indicate which entity trained you ________

**Component three: knowledge**

*Rate each aspect according to your opinion on the proposed scale: 1 strongly disagree; 2 disagree; 3 neutral (neither agree nor disagree); 4 agree; and 5 strongly disagree.* *agreement* .

| Knowledge | 1* | 2 | 3 | 4 | 5** |
| --- | --- | --- | --- | --- | --- |
| Animal welfare isn´t everything that humans provide for animals. |  |  |  |  |  |
| Stress in birds during poultry industry processes affects the quality of eggs and meat. |  |  |  |  |  |
| The presence of pododermatitis and the integrity of the keel bone are related to the quality of the environment. |  |  |  |  |  |
| When a bird exhibits severe feather plucking , it should be euthanized . |  |  |  |  |  |
| It is considered that beak correction in birds shouldn´t be done without exception. |  |  |  |  |  |
| Staff handling birds should be trained in essential aspects of animal welfare. |  |  |  |  |  |
| The dirtiness of the plumage is related to the state of the environment and the production system. |  |  |  |  |  |

**Component four: attitudes**

*Rate each aspect according to your opinion on the proposed scale: 1 strongly disagree; 2 disagree; 3 neutral (neither agree nor disagree); 4 agree; and 5 strongly disagree.* *agreement* .

| Attitudes | 1* | 2 | 3 | 4 | 5** |
| --- | --- | --- | --- | --- | --- |
| Birds feel pain. |  |  |  |  |  |
| The well-being of birds is just as important as that of human beings. |  |  |  |  |  |
| Birds should express their behaviors and conduct in a natural way. |  |  |  |  |  |
| Birds in the poultry industry should be free from fear and distress regardless of the context in which they are raised. |  |  |  |  |  |
| Birds feel preference, motivation, or aversion according to the stimuli offered by their environment. |  |  |  |  |  |
| It is important to assess the well-being of the birds every day, using an appropriate instrument. |  |  |  |  |  |
| The assessment of animal welfare in birds shouldn´t be delegated to external staff or a consultant. |  |  |  |  |  |
| A hen or a chicken can potentially or in certain circumstances be or be considered a pet. |  |  |  |  |  |

**Component five: perceptions**

*Rate each aspect according to your opinion on the proposed scale: 1 strongly disagree; 2 disagree; 3 neutral (neither agree nor disagree); 4 agree; and 5 strongly disagree.* *agreement.*

| Perceptions | 1* | 2 | 3 | 4 | 5** |
| --- | --- | --- | --- | --- | --- |
| The application of animal welfare standards has a positive impact on the health of birds and the productivity of the industry. |  |  |  |  |  |
| It is correct and appropriate to promote the application of welfare standards in the poultry industry. |  |  |  |  |  |
| Controlling environmental factors such as temperature, humidity, and ventilation in farms ensures the well-being of the birds. |  |  |  |  |  |
| Physical health, good mental state, and natural bird behavior are essential factors in animal welfare. |  |  |  |  |  |
| There is sufficient information in Colombia about animal welfare in the poultry industry. |  |  |  |  |  |
| New legislation is needed to prevent suffering in the handling of birds. |  |  |  |  |  |
| The information received from the producer is sufficient to determine the welfare conditions of the birds in the industry. |  |  |  |  |  |
| The density of birds housed on the floor or in cages must be included within the animal welfare criteria in the industry. |  |  |  |  |  |

**Component six: Opportunities for improvement on animal welfare in the poultry industry.**

*Select 3 of the answers you consider appropriate from the list.*

1. What do you consider to be the main barriers to the implementation of animal welfare standards in the Colombian poultry industry?

- Difficult access to training.
- Lack of knowledge on the part of professionals.
- The lack of motivation for economic investment on the part of the producers.
- The lack of sufficient incentives for producers.
- Beliefs about declining productivity and economic gains.
- The difficulty in arranging and planning visits to poultry farms.
- Resistance to change.
- The impact on profitability due to the decrease in density in the sheds.

2. Which of the following aspects do you think can improve the readiness for the implementation of animal welfare standards in the poultry industry?

- The creation of economic incentives for producers.
- The willingness of certain consumers to pay more for meat and eggs that meet animal welfare standards.
- The ease of access to training for producers.
- Evidence on the relationship between the implementation of animal welfare practices and economic gains.
- The best training for professionals.
- Timely, frequent and conscious visits by professionals to production units, with animal welfare criteria.
- Assertive and proactive communication by professionals regarding their concerns about animal welfare factors.
- The conviction on the part of producers regarding the need for professionals to have the knowledge and ability to implement animal welfare.

3. What limitations do you think might be encountered when promoting the adoption of animal welfare standards among producers?

- Personal lack of knowledge of the standards and their application.
- The limited ability to influence, as a professional, the decisions of the producers.
- Deeply ingrained habits among producers prevent substantial changes on this issue.
- The producer's lack of confidence in the professional's knowledge.
- Deeply rooted practices in professional practice that prevent a change in attitude.
- The producers' rejection of this issue stems from their view that the change in the number of animals per production area is negative.
- The lack of communication and trust between the professionals and the producer.
- The perception of animal welfare as something that is not essential in production.

*Thank you in advance for your participation. The information gathered here will be of great help in expanding knowledge about animal welfare in the Colombian poultry industry.*
